# Omicron subvariant BA.5 is highly contagious but containable: Successful experience from Macau

**DOI:** 10.3389/fpubh.2022.1029171

**Published:** 2023-01-10

**Authors:** Cong Xu, Jue Wang, Lili Yu, Xinbing Sui, Qibiao Wu

**Affiliations:** ^1^Faculty of Chinese Medicine, University Hospital, State Key Laboratory of Quality Research in Chinese Medicine, Macau University of Science and Technology, Taipa, Macao SAR, China; ^2^College of Pharmacy, Hangzhou Normal University, Hangzhou, Zhejiang, China; ^3^Department of Medical Oncology, The Affiliated Hospital of Hangzhou Normal University, Hangzhou Normal University, Hangzhou, Zhejiang, China; ^4^Zhuhai Macau University of Science and Technology (MUST) Science and Technology Research Institute, Zhuhai, Guangdong, China; ^5^Guangdong-Hong Kong-Macao Joint Laboratory for Contaminants Exposure and Health, Guangdong University of Technology, Guangzhou, China

**Keywords:** successful experience, Macau, Omicron subvariant BA.5, “relatively static” strategy, anti-COVID-19, containable

## Abstract

**Introduction:**

Due to its high transmissibility and immune escape, Omicron subvariant BA.5 has become the dominant strain of the SARS-CoV-2 virus and led to escalating COVID-19 cases, how to cope with it becomes an urgent issue. A BA.5 infection surge burst out on 18 June 2022 and brought an unprecedented challenge to Macau, the most densely populated region worldwide. This study aimed to analyze the characteristics of this outbreak and summarize the useful anti-epidemic measures and experiences during this outbreak.

**Methods:**

All data were obtained from the Government Portal of Macao SAR (https://www.gov.mo), and the Special Webpage Against Epidemics, the Macao Health Bureau (www.ssm.gov.mo). An epidemiologic study was performed to analyze epidemic outcomes, including the infection rate, the proportion of symptomatic cases, the case fatality ratio (CFR), etc. Data were analyzed using SPSS Version 20. A *p*-value <0.05 was considered statistically significant. The anti-epidemic measures and experience were reviewed and summarized.

**Results:**

The BA.5 outbreak resulted in 1,821 new cases, which was significantly more than the cumulative cases of the previous variants of COVID-19 in Macau. The symptomatic cases accounted for 38.71% of the total cases, which was higher than that of the previous variants. After 6-week concerted efforts, Macau effectively controlled the outbreak, with an infection rate of 0.27%, which was much lower than many BA.5-attacked regions. The CFR was approximately 0.86%, which was not statistically different from that of previous variants. Six victims were chronically ill senior elders and their vaccination rate was much lower than the average level. Macau took a comprehensive anti-epidemic strategy to win a quick victory against BA.5, especially the “relatively static” strategy that was first formulated and applied by Macau for the management of the COVID-19 pandemic. Successful experience showed that although BA.5 was highly contagious, it could be contained by comprehensive anti-epidemic measures, including adequate anti-epidemic preparation, herd immunity through vaccination, repeated mass nucleic acid tests and rapid antigen tests, KN-95 mask mandate, the “relatively static” strategy, precise prevention and control, epidemiological investigation and tracing, and traditional Chinese medicine treatment, etc.

**Discussion:**

In Macau, compared with the previous subvariants, BA.5 is associated with increased transmissibility and a higher proportion of symptomatic cases, however, the risk of death remains similar, and the infection rate is much lower than that in many other BA.5-attacked regions. BA.5 is highly contagious but still containable, Macau's experience may offer hints for the regions experiencing the BA.5 waves to choose or adjust a more rational anti-epidemic strategy.

## 1. Introduction

The BA.5 variant of Omicron was first detected in February 2022 in South Africa (1, 2). Currently, it has been spreading rapidly throughout the world and leads to a significant rise in new cases and health concerns amid the ongoing wave of the COVID-19 pandemic (2, 3). Compared with previous variants (including the Alpha, Beta, Gamma, and Delta variants of SARS-CoV-2, and the BA.1, BA.2., BA.3, BA.4 lineages of Omicron, etc.) (4–7), the latest BA.5 variant is better at evading the immune system and has become the most easily transmissible variant to date (5, 8). It has gradually become the dominant strain of the SARS-CoV-2 virus in many parts of the world and has been associated with the rapidly escalating COVID-19 cases and hospitalizations.

In the U.S., BA.5 has accounted for an estimated 80% of new cases (around 100,000/day), and the number continues to grow (9, 10). The coronavirus hospitalization rate in New York has boomed by 70% in the past month (11). In Europe, the rapid spread of Omicron subvariants (BA.4 and BA.5) has also contributed to a summer surge of COVID-19. New cases have tripled and the hospitalization rates doubled over the past 6 weeks. New cases rose to 3 million in a week, accounting for nearly 50% of global new cases. There will be nearly 3,000 COVID-19 deaths every week due to the rising infection rate in older groups (12, 13). BA.5 is more than four times as vaccine-resistant as its predecessors and appears to be able to infect individuals who have been previously vaccinated and boosted against COVID-19 or have been previously infected with COVID-19 or even both. Due to the characteristics of BA.5 with strong concealment and fast transmission speed, how to cope with it becomes an urgent issue (11–15).

On 18 June, a new outbreak of COVID-19 occurred in Macao Special Administrative Region (SAR), China. As of 28 July, a total of 1,821 new COVID-19 cases have been recorded. Fortunately, adhering to the principles of scientific and precise prevention and control, Macau took a series of timely and effective anti-epidemic measures in response to epidemic changes (16). After 5-week concerted efforts, the spread of the most transmissible variant of COVID-19 has been effectively minimized, the outbreak has been successfully controlled, approaching the ultimate goal of “dynamic zero-COVID-19 strategy” in the community (17).

The COVID-19 Omicron subvariant BA.5.1 was the culprit of this largest spike in infections since the emergence of COVID-19 in Macau. To the best of our knowledge, so far, Macau is the first region in the world that took the “relatively static” control measures and active anti-epidemic strategy to win the battle against the BA.5 epidemic in a very short period (40 days), thus keeping the epidemic under control and resuming the normal socioeconomic activities as soon as possible, successful experience from Macau shows that timely and effective anti-epidemic measures, especially the “relatively static” strategy, may offer hints about the future prevention and control of the BA.5 subvariant, although BA.5 is highly contagious, insidious, and daunting, it can be contained (18).

## 2. Methods

All data were obtained from the Government Portal of Macao SAR (https://www.gov.mo), and the Special Webpage Against Epidemics, the Macao Health Bureau (www.ssm.gov.mo).

An epidemiologic study was performed to analyze the outcomes of the epidemic, including the infection rate of the total population, the proportion of symptomatic cases or asymptomatic cases, and the case fatality ratio among the total BA.5 subvariant cases, etc. The differences between BA.5 and the previous variants were compared. Data were analyzed using SPSS Version 20. A *p*-value < 0.05 was considered statistically significant. The anti-epidemic measures and experience from Macau during this outbreak of the BA.5 subvariant were reviewed and summarized.

## 3. Results

### 3.1. The facts of Omicron subvariant BA.5 in Macao SAR

**Figure 1:**
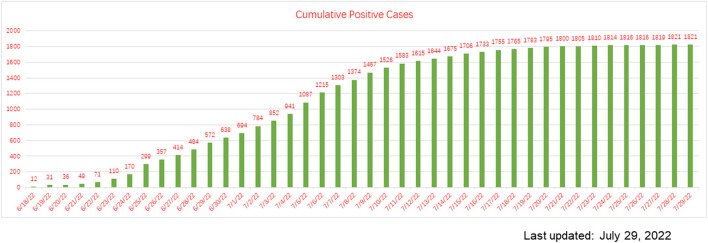
Cumulative positive cases during the BA.5 subvariant COVID-19 outbreak in Macau (data from the news bulletin of the Macao Health Bureau, was last updated on July 29, 2022).

In the past 2½ years, the cumulative number of COVID-19 cases in Macau was only 335 (the total population: 683.2 thousand), but a BA.5 infection surge burst out on 18 June 2022, resulting in 1,821 new cases, with six deaths, as of July 28 when the last positive case of this outbreak was reported (Figure 1) (17), which was significantly more than the cumulative cases of the previous variants of COVID-19 in Macau, suggesting that BA.5 is more transmissible than the previous subvariants. The symptomatic cases accounted for 38.71% of the total BA.5 subvariant cases, which was higher than that of the previous variants of COVID-19 (SARS-CoV-2 virus, Delta variant, Alpha variant, Omicron BA.1, etc.; 25.67%) in Macau (Table 1) (17). The COVID-19 vaccination rate in Macau was 90.04%, and most positive cases were fully vaccinated, but still infected with BA.5. The facts of Omicron subvariant BA.5 in Macau are consistent with its known characteristics: more transmissible and immune-evading, even people who are fully vaccinated are likely still at risk for BA.5.

**Table 1:** Clinical outcomes of the positive cases with BA.5 or previous subvariants.

**Outcomes**	**Previous subvariants**	**BA.5 subvariant**	***P*-value**
	***n* (%)**	***n* (%)**	
Symptomatic cases	86 (25.67%)	705 (38.71%)	< 0.0001
Asymptomatic cases	249 (74.33%)	1,116 (61.29%)	
Case fatality ratio (CFR)	0 (0.00%)	6 (0.86%)	0.84

Data from the news bulletin of the Macao Health Bureau, was last updated on July 29, 2022.

The Case Fatality Ratio (CFR) of the BA.5 subvariant of COVID-19 was ~0.86%, which was not statistically different from that of previous variants, suggesting that the risk of death seemed similar between the BA.5 and previous variants (Table 1) (17). All six victims of the BA.5 subvariant were chronically ill senior elders (over 88 years old) and their vaccination rate was much lower than the average level (Table 1) (17). All the patients had a history of chronic diseases, such as heart and lung diseases, brain degeneration, fractures, hyperlipidemia, stroke, and other chronic underlying diseases (Table 2). The facts reminded us again that chronically ill elders are the most vulnerable population during the pandemic and they need focused protection and should get vaccinated or boosted as soon as possible.

**Table 2:** The characteristics of six COVID-19 victims.

**Number**	**Age**	**Sex**	**Status of COVID-19 vaccination**	**Previous medical status**
1	94	Female	Had received two doses of inactivated COVID-19 vaccines.	She had a history of hypertension, hyperlipidemia, stroke, and other chronic underlying diseases, she required long-term care.
2	100	Female	Not vaccinated against COVID-19.	She had a history of hypertension, brain degeneration, fractures, and other chronic underlying diseases; she was bedridden and in need of long-term care.
3	88	Female	Not vaccinated against COVID-19.	She was bedridden and in need of long-term care, she had a history of severe diabetes mellitus, heart disease, aortic dissection, etc.
4	94	Female	Not vaccinated against COVID-19.	She was dependent on caregivers, had no self-care ability, and suffered from chronic heart and respiratory failure.
5	86	Female	Had received two doses of inactivated COVID-19 vaccines.	She suffered from chronic renal disease, gastrointestinal bleeding, and other chronic underlying diseases.
6	93	Male	Not vaccinated against COVID-19.	He suffered from chronic heart disease and chronic pulmonary disease and required long-term oxygen therapy at home.

### 3.2. Successful experience from Macao SAR

**Figure 2:**
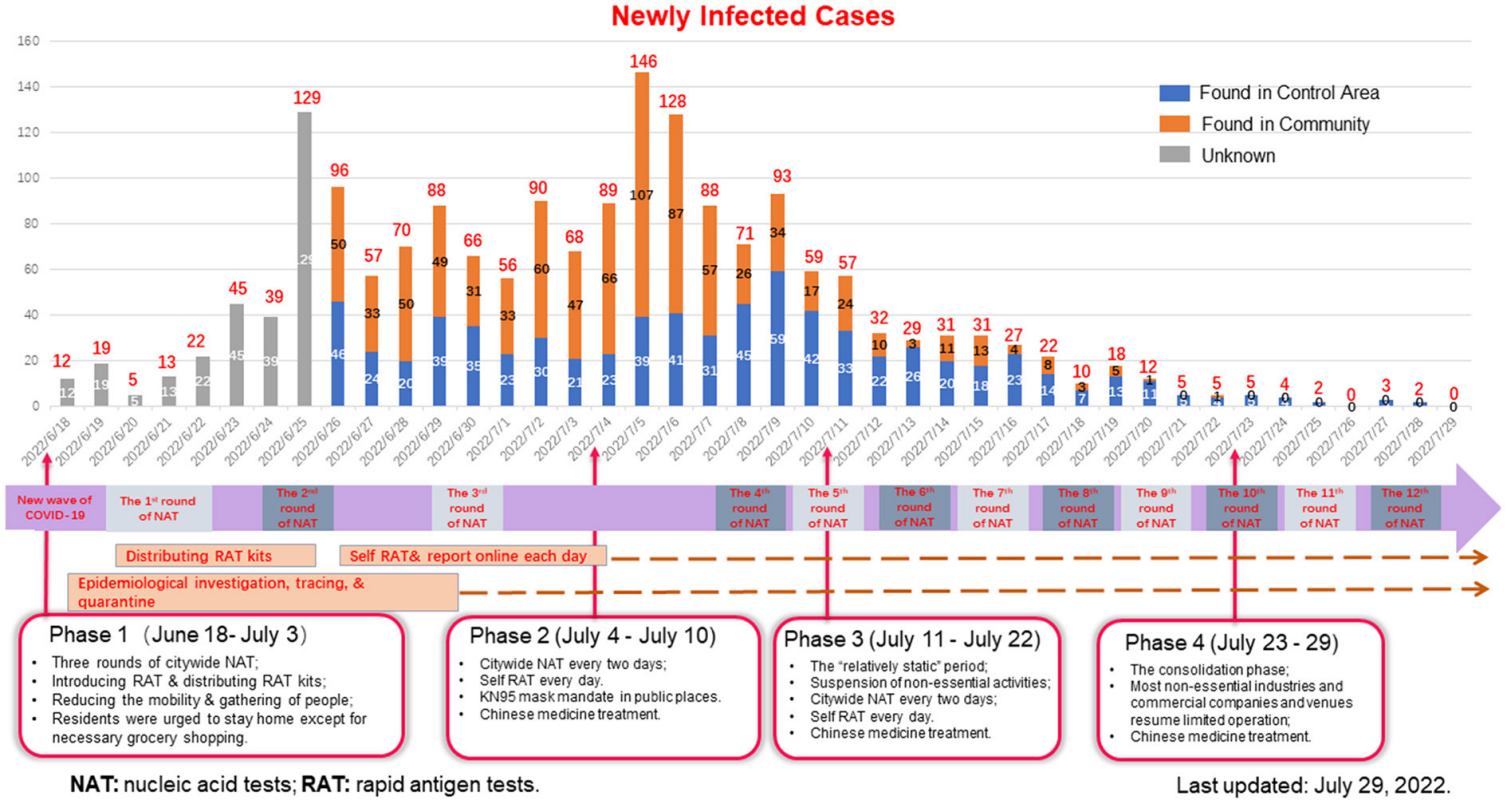
Daily positive cases and dynamically adjusted measures during the BA.5 subvariant COVID-19 outbreak in Macau (data from the news bulletin of the Macao Health Bureau, last updated on July 29, 2022).

In response to this epidemic, the Macao SAR government assessed the epidemic development scientifically and adopted a series of comprehensive measures to combat the pandemic, meanwhile keeping the balance between minimizing the virus transmission and ensuring the socio-economic operation and the essential living needs of residents (17). BA.5 brought an unprecedented challenge to Macau, the most densely populated region worldwide. Fortunately, after 6-week concerted efforts, Macau effectively controlled the outbreak, with an infection rate of 0.27% (1,821/6,832,000), which was much lower than that in some BA.5-attacked regions, such as the US and Europe (9–13). Macau took a comprehensive anti-epidemic strategy to win a quick victory against BA.5, especially the “relatively static” strategy that was first formulated and applied by Macau for the management of the COVID-19 pandemic. Macau won the battle against the BA.5 epidemic in a very short period (40 days). Successful experience shows that although BA.5 is highly contagious, it can be conquered by comprehensive anti-epidemic measures, including adequate anti-epidemic preparation, herd immunity through vaccination, repeated mass nucleic acid tests and rapid antigen tests, KN-95 mask mandate, the “relatively static” strategy, precise prevention and control, epidemiological investigation and tracing, etc. The comprehensive measures are as follows, and are roughly summarized in Table 3 and Figure 2 (17).

**Table 3:** The comprehensive measures dynamically adjusted by Macau to combat the pandemic.

**Phase**	**Features**	**Measures (14)**
Phase 1 June 18–July 3	A new wave of COVID-19 burst out, and new cases increased every day.	1. The SAR Government announced that Macao SAR went into an immediate state of prevention starting from 1:00 a.m. on 19 June 2022; 2. Updated the conditions for border crossing; 3. Three rounds of citywide nucleic acid tests (NAT); 4. NAT administered for key groups and areas; 5. Rapid antigen tests (RAT) were introduced and RAT kits were distributed. 6. The public entertainment venues such as cinemas, beauty salons, gymnasiums, and bars were closed, as well as provision of dine-in services in all food and beverage establishments has been suspended. 7. The Government urged all residents to stay at home, and avoid going out, except for necessary grocery shopping.
Phase 2 July 4–10	New cases increased every day with a peak of 146 new positive cases on July 5.	1. Repeated citywide NAT, with each round finished in 2 days (36 h); 2. Self-RAT every day and results were reported online; 3. N95 masks were distributed by the government, and wearing N95 masks when in a public area became a mandatory requirement; 4. A mobile sampling team was set up to conduct on-site sampling for those tested positive in mixed samples, all these measures effectively reduced the risk of cross-infection and the daily local cases; 5. Traditional Chinese medicine treatment was used for patients based on their consent and demands.
Phase 3 July 11–22	The proportion of new positive cases in the community was high, and the “relatively static” control measures were taken to prevent the wide spread of BA.5 in communities.	1. Repeated citywide NAT, with each round finished in 2 days (36 h); 2. Self-RAT every day and results were reported online. 3. Suspension of non-essential industries and commercial activities. 4. The “stay-at-home” mandate. 5. Exemptions applied to three categories of activity deemed essential to the community and to the day-to-day lives of the members of the public, including the companies providing basic public services, services deemed necessary for the daily lives of the members of the public, those companies, entities, and venues that have received approval to continue operations from their respective supervising authorities.
The consolidation period. July 23–29	The number of daily new cases was close to zero in the community. Aimed to enable people to gradually return to their normal life.	1. Limited operation of non-essential industries and commercial activities to resume; 2. Public departments provide limited public services; 3. The lowest possible level of non-essential movement in the community; 4. An extra NAT drive was further carried out in the key areas to identify any potentially infected persons who might still be lurking in the local community, people in the key areas were subject to a NAT daily. 5. The public could walk their dogs out under the prerequisite of compliance with the anti-epidemic requirements.

The region-specific, multi-level epidemic prevention and control was launched during all phases: the relevant buildings were classified as “lockdown zones,” “red” health codes, and on-site NATs were applied to the individuals there, epidemiological investigation, tracing, and quarantine, etc. (14).

#### 3.2.1. Adequate anti-epidemic preparation in advance

In case of a large-scale outbreak, the SAR government keeps promoting the precise prevention and control level through science, continuously perfecting anti-epidemic measures, and making adequate preparation in advance. In April 2022, the SAR government prepared and formulated a 118-page “The Contingency Plan Regarding Large-scale COVID-19 Outbreak” to enact a full and rapid deployment and response in case of a large-scale COVID-19 outbreak in Macau (16). The Contingency Plan was released to the public in different languages, and drills were organized, therefore, when the BA.5 epidemic burst out, the well-prepared government, health professionals, social organizations, volunteers, and residents could respond calmly, quickly, and methodically.

Besides, the SAR government prepared and provided free adequate anti-epidemic supplies and services, such as the efficient citywide nucleic acid testing (NAT) capability, sufficient isolation and treatment facilities, information and communication outlets, epidemiological tracing capacity, the KN-95 masks, rapid antigen test (RAT) kits, etc., which laid a solid foundation for the victory in fighting the epidemic.

#### 3.2.2. High COVID-19 vaccination rate

The WHO goal was to achieve 70% COVID-19 immunization coverage by June 2022, which remains a daunting challenge due to limited vaccine supply to some regions (19). In Macau, the COVID-19 vaccination rate has reached 90.04%, and over 86% of residents have received a second COVID-19 booster shot (20), indicating that Macau residents have achieved herd immunity against COVID-19 before this BA.5 outbreak, which might be one of the important reasons why most infected cases during the outbreak were asymptomatic (61.39%), there were very few hospitalizations or severe cases, and only six victims. The six victims were chronically ill senior elders (over 88 years old) and 4 of them did not get vaccinated (Tables 1, 2). These facts were consistent with the previous findings, although BA.5 is more adept at slipping past immune defenses, vaccination is still the best way to reduce the risks of infection, hospitalization, severe illness, and mortality (21).

Common side effects of the COVID-19 vaccines and statistics of Adverse Events Following Immunization (AEFIs) in Macau were presented in Tables 4, 5. The vaccines have succeeded in preventing severe disease or death, but more novel next-gen COVID-19 vaccines are still necessary for keeping people from catching and spreading the virus because SARS-CoV-2 has become more contagious and it is evolving to dodge the vaccines (7, 22, 23).

**Table 4:** Statistics of Adverse Events Following Immunization (AEFIs).

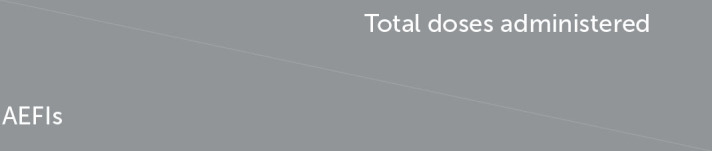			**Inactivated vaccine (Sinopharm)**	**BioNTech mRNA vaccine (BioNTech)**	**Other vaccines (non-local administration)**	**Total**
			**1,370,040**	**309,058**	**4,704**	**1,683,802**
1. Minor adverse events		1.1. Reaction caused by the vaccine	2,266	1,717	N.R.	3,983
		1.2. Immunization error-related reaction	0	0	N.R.	0
		1.3. Immunization Anxiety-related reaction	22	11	N.R.	33
		1.4. Coincidental event or uncertain	1,101	370	N.R.	1,471
2. Serious adverse events	2.1. Anaphylaxis		1	0	N.R.	1
	2.2. Other serious adverse events	2.2.1. Reaction caused by the vaccine	0	N.R.	N.R.	4
		2.2.2. Immunization error-related reaction	0	0	N.R.	0
		2.2.3. Immunization anxiety-related reaction	0	0	N.R.	0
		2.2.4. Coincidental event or uncertain	3	6	N.R.	9
**Total**			**3,393**	**2,108**	N.R.	**5,501**

AEFIs, Adverse Events Following Immunization; N.R., not reported.

Data from Serviços de Saúde, Governo da Região Administrativa Especial de Macau. Weekly Bulletin on COVID-19 Vaccination and Adverse Events Following Immunization (AEFI) in Macao, Last updated: December 8, 2022.

**Table 5:** Rate of Adverse Events Following Immunization reported (per 1,000 doses).

	**Inactivated vaccine (Sinopharm)**	**mRNA vaccine (BioNTech)**	**Total**
Serious adverse events	0.003	0.033	0.009
Minor adverse events	2.665	6.926	3.484
Total	2.668	6.959	3.493

Serious adverse event: if it results in death, is life-threatening, requires in-patient hospitalization or prolongation of existing hospitalization, results in persistent or significant disability/incapacity, is a congenital anomaly/birth defect, or requires intervention to prevent permanent impairment or damage.

Data from Serviços de Saúde, Governo da Região Administrativa Especial de Macau. Weekly Bulletin on COVID-19 Vaccination and Adverse Events Following Immunization (AEFI) in Macao, Last updated: December 8, 2022.

#### 3.2.3. The efficient citywide nucleic acid test (NAT) capability

The SAR government kept improving the NAT capacity, the efficient mass NAT capability ensured that each round of citywide NATs for about 680,000 residents could be implemented every 2 days (usually in 36 h) (16, 17). Anyone who refused to take the NAT test would be sent to medical observation for 14 days. Vast numbers of volunteers and civil servants in Macau have participated in the epidemic prevention work with selfless dedication, residents and different sectors of the society have actively cooperated, and frontline anti-epidemic staff have been committed to their duties and responsibilities without complaint. The support of 650 samplers from mainland China significantly enhanced Macau's NAT capability (17).

#### 3.2.4. KN-95 masks

The SAR government distributed KN-95 masks to all residents and it was a mandatory demand that members of the public should wear a KN95 mask at all times when going out or at workplaces (24). KN95 Masks can filter 95% or more airborne particles, a higher filtration rate for fine particles as compared with surgical masks, thus providing sufficient protection against the SARS-CoV-2 Omicron variant (25).

#### 3.2.5. Daily rapid antigen test (RAT)

All people in Macau were required to take RATs every day and report the result *via* the “Macau Health Code app” (26). Self-RAT at home could reduce the mobility of people, the risk of transmission in the community, and the burden on medical and public health services. If the test result was declared as positive, the health code would be converted to a “red code,” one should call an ambulance to undergo a NAT as soon as possible. In addition, if one had a positive RAT, his/her roommates were not allowed to go out, and a nucleic acid test would be arranged for all of them by the authorities (26).

#### 3.2.6. Precise prevention and control by zones and levels

Macau abided by a “zone-specific, multi-level targeted approach to epidemic prevention and control,” classified buildings with positive cases as medium or high-risk areas, and promptly implemented precise prevention and control measures according to the levels of risk. Areas were classified as “red-coded zones” and “yellow-coded zones,” with a tentative quarantine period of 7 days. Comprehensive restriction and closure management were implemented. The government catered to the daily living needs of people in the red- or yellow-coded zones (27).

#### 3.2.7. The “relatively static” control measures

In the mainland of China, a strict lockdown plays a crucial role in the successful control of serious outbreaks of the pandemic, however, which demands huge manpower to ensure that every household in the lockdown areas gets the supply of the necessaries of life, and the precise street- or community-based committees are essential at the operation level. Similar to most countries or regions, Macau lacks the logistics support capacity, and it was almost impossible to implement a strict lockdown in Macau. Therefore, when the proportion of community infections remained high, the SAR government took the “relatively static” control management in the third stage, intending to prevent the wide spread of BA.5 in communities (17, 18).

The “relatively static” strategy (or a relative lockdown) was a unique anti-epidemic measure that Macau adopted according to its situation (a strict lockdown was impossible), which played a key role in the control of the BA.5 outbreak in Macau, measures were adopted to reduce unnecessary movement and gathering of people, to break the chains of virus transmission as soon as possible, and create necessary conditions for achieving “dynamic zero-COVID-19.” With the region-specific approach, the lockdown area was defined as small as possible, usually limited to buildings, and the residents outside of the lockdown area were ordered to stay home when community infections rapidly increased, while short trips for essential services, necessaries of life were allowed, for example, going to supermarkets or drugstores for daily necessities or medicines when necessary, thus keeping the balance between adopting rigorous pandemic measures and satisfying the basic demands of residents (17, 18).

The implementation of the “relatively static” measure inevitably brought some inconvenience to normal work and life. But, almost all residents could fulfill their civic responsibility, and strictly comply with the relevant laws and regulations on epidemic prevention, contributing to fighting against the epidemic. Since the implementation of “relatively static” management measures on 9 July, the number of new daily positive cases was gradually decreasing, with 10 cases on July 18, 2022. These results suggested that the “relatively static” measures were effective, contributing to achieving the “dynamic zero” goal (17, 18).

#### 3.2.8. The efficient epidemiological investigation and tracing capacity

Macau continues to reinforce and upgrade the epidemiological investigation and tracing capacity, including establishing and perfecting the epidemiological contact tracing database, recruiting and training epidemiological investigation staff, rolling out the “Macao Health Code” Mobile App to receive NAT or RAT reports and record itinerary and risk assessment, posting venue QR codes for residents to scan upon entering to record their itineraries, enhancing the collaboration with the neighboring areas, thus tracing the contacts of any positive cases and its source of infection, determining the chain of infection and risk sites, making recommendations on public health measures, and arranging further examination for various risk groups (17, 18).

#### 3.2.9. Traditional Chinese medicine (TCM) treatment

Evidence-based medicine has confirmed that TCM combined with western medicine may have clinical advantages for COVID-19 patients, such as alleviating symptoms, improving prognosis, etc. (28, 29). WHO Expert Meeting on Evaluation of TCM in the Treatment of COVID-19 recommended Member States consider the potential use of TCM for the management of COVID-19 (29). During this outbreak, the TCM practitioners actively volunteered to participate in the Chinese Medicine Anti-epidemic Team and provided consultations and guidance to the residents. Two hundred and seventy-five TCM practitioners participated in the treatment process and gave medication guidance. Finally, 74.14% (1,500/1,821) positive cases including some foreign nationals chose to receive TCM treatment and took Chinese patent medicines (30), Chinese patent medicines including Lianhua Qingwen capsules (29) and Huoxiang Zhengqi soft capsules / oral solution (31, 32) were most commonly prescribed for the patients based on their consent, which greatly facilitated to improve symptoms and promote recovery (28–32). Two hundred and sixty-eight out of the 408 (67%) foreign patients also chose to receive TCM treatment.

## 4. Discussion and conclusion

The mutations of novel variants facilitate the virus to dodge the immune of individuals with either vaccination or previous infection, the emergence of new variants, such as BA.5, has posed an increased risk to global public health (15, 33). Macau is the most densely populated region in the world, and the highly contagious Omicron subvariant BA.5 posed an unprecedented challenge to the prevention and treatment of COVID-19 in Macau. Macau could not choose to “lie flat” policy, on the other hand, it was extremely difficult for Macau to enforce a strict lockdown.

Since the pandemic broke out, divides have emerged about the right path out of COVID-19 (34). Currently, most countries have chosen to co-exist with coronavirus, or so-called “lying flat,” which means that epidemic control measures are relaxed, mask mandates, mass COVID-19 testing, social distancing, or quarantine is not required. But, along with the worldwide spread of BA.4 or BA.5, many countries or cities are seeing rapid spikes in COVID-19 cases, globally, there are over 6,700,000 new cases and 12,000 deaths in a week (35). Easing coronavirus policies is one of the reasons for the surge of COVID-19 new cases and hospitalizations in the relevant regions. WHO Director-General Tedros Adhanom Ghebreyesus recently expressed his concern that many countries are drastically reducing epidemic control measures (36).

According to their own situation, a few countries/regions think “lying flat” policy may bring disaster to vulnerable populations and overwhelm the healthcare system, therefore they choose to hold on to the “dynamic zero-COVID-19 strategy” goal, which is usually achieved by the strict lockdown measures, but, it is very difficult to enforce a strict lockdown and may cause counterproductive damage to the economy and living of the public (17).

Successful experience from Macao SAR indicates that Omicron subvariant BA.5 is highly contagious but still conquerable, which can be prevented and controlled by comprehensive measures including adequate preparation, vaccination, mass NAT and RAT, KN-95 masks, precise prevention and control, epidemiological investigation and tracing, TCM treatment, etc. Among them, the “relatively static” strategy is crucial for the success of epidemic control in Macau and may provide a new choice for the global fight against subvariant BA.5, the “relatively static” strategy is a middle way between “lying flat” and lockdown, aiming to strike a balance between pandemic control and enabling residents to live normal lives. The “relatively static” strategy is a unique anti-epidemic measure that Macau took according to its situation and based on the experience of other regions, and it has been proven as effective as a strict lockdown to achieve “dynamic zero-COVID-19 strategy-19” in the community even in a COVID-19 spike caused by the most contagious Omicron BA.5 (17, 18). Macau's experience may offer hints for the regions experiencing the BA.5 waves. The regions experiencing the latest variant waves may be encouraged and inspired by Macau's success and experience, thus choosing or adjusting a more suitable strategy.

Considering that the emergence of the latest Omicron variants has caused a serious situation, and the rapidly spreading viruses have chances to mutate into possibly more devastating variants, it might be necessary for regions that already removed their anti-pandemic measures to reflect and re-evaluate their COVID-19 policies. On the other hand, increasing evidence has suggested that the Omicron variants appear to be more contagious but less deadly than other lineages of the virus, with the potential decrease in toxicity of new Omicron variants and the increasing COVID-19 vaccination rate, in the long run, for those regions that hold on to a strict lockdown policy, there is also a need or a tendency to gradually relax the strict measures, thus promoting the recovery of the normal socioeconomic activities and international exchanges, but it must be a long process of exploration, Macau's experience also provides some perspective for these regions in decision-making on the strategy against the pandemic.

## Data availability statement

The original contributions presented in the study are included in the article/supplementary material, further inquiries can be directed to the corresponding authors.

## Author contributions

CX: conceptualization, investigation, methodology, analysis, and writing an original draft. LY and JW: investigation, validation, formal analysis, and writing and editing. XS: validation, investigation, and formal analysis. LY, XS, and QW: conceptualization, supervision, methodology, and review and revision of the draft. All authors contributed to the article and approved the submitted version.
